# Child Who Presented with Facial Hematohidrosis Compared with Published Cases

**DOI:** 10.1155/2016/5095781

**Published:** 2016-03-14

**Authors:** Ali Jafar, Ali Ahmad

**Affiliations:** ^1^Department of Surgical & Interventional Sciences, Division of Surgical and Interventional Sciences, Royal Free Hospital, University College London (UCL), London NW3 2QG, UK; ^2^Department of Internal Medicine, Mubarak Al-Kabeer Hospital, Jabriya, Kuwait

## Abstract

Hematohidrosis is a rare condition in which an individual sweats blood from intact, unbroken skin. The aetiology of hematohidrosis is not clear, although various theories exist to explain such a phenomenon. The general consensus however in the literature relates the finding to dermal capillary blood vessels that rupture under extreme emotional or physical stress exuding blood through the skin. In this case report we disclose a case of 12-year-old girl who presented with unusual painless bleeding from her face, eye, and tear duct. The condition was investigated intensively during hospital admission for a cause, and no actual cause was speculated. The management mostly involved supportive care and medical advice.

## 1. Case Presentation

**Figure 1:**
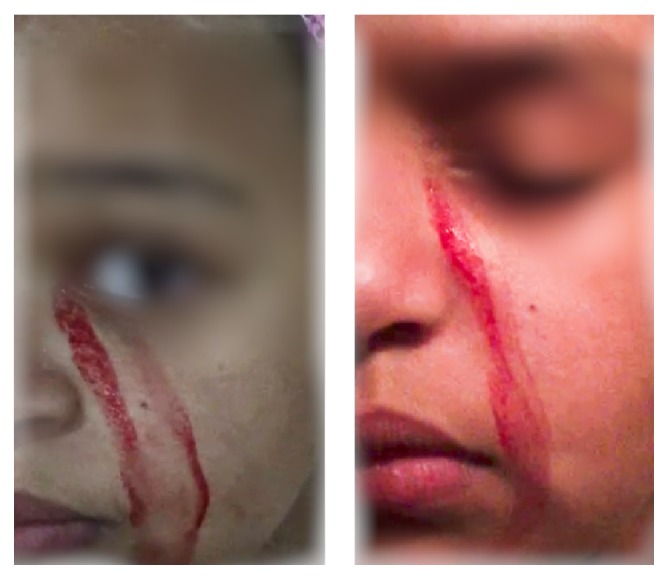
Unilateral hematohidrosis of left facial side observed on two separate occasions.

A 12-year-old girl was admitted to the hospital as a case of unusual painless bleeding from the left side of the face (Figure 1), left eye, and tear duct of two-week duration. The bleeding was described to be spontaneous, unpredictable, and intermittent, with no specific patterns, and it stopped spontaneously. There was no association with mood, activity, or sleep. The bleeding occurred mostly around the orbital regions, the tear ducts, and the face, with intact skin, and no blood or redness, or visual disturbance of the eyes, except for mild perorbital tenderness. Several episodes of bleeding occurred prior to admission, and on admission two episodes occurred while the physician observed. Each episode started like tear drop confluent spots of mild watery secretion over the left side of the face and streak-like drops starting from the corner of eye where tear duct is situated and along the cheeks. This was followed by bright-red colored secretion, lasting for 10–20 minutes, and the patient did not show any signs of distress, siting comfortably in the bed during the episodes. The patient describes occasional gum bleeds when brushing teeth, although she denied hematuria, blood in stools, or blood stained clothes or underwear, and there was no evidence of petechiae, bruising, or ecchymosis. During this admission, the patient had one episode of epistaxis, which was treated with cautery. However, previously, the patient had presented twice in June 2015 with two episodes of epistaxis and on both occasions was treated with cautery.

CT scan was performed on the patient to be assessed for any sinus disease and showed mucosal thickening in bilateral maxillary sinuses and sinus orifice obstruction consistent with chronic sinusitis. Additionally CT scan showed bony nasal septal deviation to the right.

Previous admission was on 2013 (2 years ago) for repeated faint attacks, palpitations, and mild anemia, with normal ECG findings.

On system review, patient denied fever, runny itchy nose, nasal discharge, red or painful eye, shortness of breath, cough, chest pain, palpitations, rash, joint pain, mouth ulcers, bleeding PR/urine, menorrhagia, abdominal pain or change in bowel habits, anorexia/recent weight lose, sensory loss or weakness, and lumps or bumps or lymphadenopathy. Her physical examination was unremarkable, except for right nasal septum deviation, enlarged adenoids, hypertrophied bilateral inferior turbinates, and mild periorbital tenderness. Complete blood count (CBC), renal function test (RFT), liver function test (LFT), and coagulation profile were all normal.

The secretion at the corner of the eye was collected during an episode of bleeding and we did blood smear examination on the sample. The results showed plenty of red blood cells, and no abnormal cells.

Hematology, dermatology, ophthalmology, ENT, and immunology were consulted to further evaluate and assess the patient, and cause of bleeding. All consults returned inconclusive of any identifiable source or cause of bleeding.

The only significant finding on past medical history included menarche at age of 10 years that was regular for four months, then spontaneously stopped for around two years, and returned spontaneously for unknown cause, with no medical attention. Last few menstruations however were regular and of normal flow and no clots or dysmenorrhea.

Patient is not on any medications and not on over-the-counter drugs. However patient describes allergy to eggs or egg-containing products and sugar candy, where a steak line rash appears on arms.

There are no family histories of such events of spontaneous bleeding amongst close relatives.

## 2. Discussion

Hematohidrosis is a rare clinical condition that manifests as self-limiting episodes of spontaneous discharge of bloody secretion through intact skin or sweat gland orifices, with an unknown cause [6]. Some theories have been proposed, including increased vascular pressure leading to the passage of blood cells through the ducts of the sweat glands, vasculitis of dermal vessels, and exacerbated sympathetic activation leading to periglandular vessel constriction and subsequent expansion, allowing the passage of blood content into ducts [7].

Few theories have been proposed regarding the etiopathogenesis of hematohidrosis [8]. One such theory describes that there are multiple blood vessels around the sweat glands arranged in a net-like form. It is believed that under the pressure of great stress the vessels contract. Subsequently as the anxiety passes the blood vessels dilate to the point of rupture. The blood at this point goes into the sweat glands, which push the blood to the surface, and manifests as droplets of blood mixed with sweat [8].

Hematohidrosis is a condition in which blood vessel capillaries that feeds the sweat glands rupture, causing them to exude blood, and occurs under conditions of extreme physical or emotional stress. Various causative and associated factors have been reported, such that vicarious menstruation, excessive exertion, psychogenic purpura, and unknown causes have been suggested [9].

The severe mental anxiety activates the sympathetic nervous system to invoke the stress-fight or flight reaction to such a degree as to cause hemorrhage of the vessels supplying the sweat glands into the ducts of the sweat glands [7, 9].

Literature search was performed to compare our case of hematohidrosis to other published cases which related closely (Table 1). The majority of the cases of hematohidrosis had spontaneous episodes of bleeding, from various locations of the body, but mostly concentrating on the facial regions (e.g., ear, nose, and eyes); no apparent aetiology could be identified although some were stress induced, no specific therapy was deemed appropriate, and resolution of symptoms occurred spontaneously. Further studies need to search for aetiology and risk factors of such condition to correctly address clinical management.

## 3. Conclusion

Hematohidrosis is a condition in which capillary blood vessels feed the sweat glands rupture, causing them to exude blood; it occurs under conditions of extreme physical or emotional stress. The treatment of hematohidrosis remains a challenge. Several options have been described such as vitamin C, hemostatic drugs, anxiolytics, or antidepressants, but none have been proven to be effective.

## Figures and Tables

**Table 1:** Literature review of cases with hematohidrosis.

Case report	Biography	Final diagnosis	Workup	Management
Da Silva Carvalho et al. (2008) [1]	A 13-year-old girl	Hematidrosis around the mouth after strenuous exercise or prolonged exposure to heat	Clinical history and physical examination were normal. Laboratory tests were normal. Skin biopsy showed a normal epidermis and a dermis with preserved pilosebaceous annexes and eccrine sweat glands. There were small, congested, periglandular blood capillaries, some in close contact, without red blood cell extravasation.	The patient evolved with spontaneous improvement of the condition.

Praveen and Vincent (2012) [2]	A 10-year-old girl	Hematidrosis and haemolacria (from forehead, neck, umbilicus, wrists, and legs) and epistaxis; all episodes of bleeding were preceded by a stressful event	General clinical examination and all laboratory evaluations were normal.	A trial of oral lorazepam in a patient with hematidrosis. However, it had to be stopped after 3 days due to intolerable side effects. She was prescribed oral propranolol and her symptoms have improved.

Tshifularo (2014) [3]	An 18-year-old girl	Bloody otorrhea induced by stressful school life	General clinical examination and all laboratory evaluations were normal.	Reassurance provided with spontaneous resolution overtime.

Biswas et al. (2013) [4]	A 12-year-old girl	Hematohidrosis from the intact skin over the forehead, scalp, cheek, nose, and trunk, without prior stressful events	Clinical history and physical examination were normal. Laboratory tests were normal. Skin biopsy was normal. Benzidine test of the secretion confirmed the presence of blood. Peripheral smear of the secretion showed RBCs and numerous cocci and bacilli.	Patient received transdermal atropine patches over the affected bleeding areas for one-month duration and gradual improvement was noted.

Deshpande et al. (2014) [5]	A 10-year-old boy	Recurrent episodes of hematohidrosis from umbilical, eyes, ear lobules, and nose regions with preexisting oppositional defiant disorder	General clinical examination and all laboratory evaluations were normal.	Therapy initiated with lorazepam during admission course and propranolol. Patient condition improved gradually and was discharged with propranolol only. However, the main focus of management during follow-up was nonpharmacological management that consisted of behavioral interventions for the child and counseling and psychoeducation to the parents.
